# Factors associated with the severity of COVID‐19 outcomes in people with neuromuscular diseases: Data from the International Neuromuscular COVID‐19 Registry

**DOI:** 10.1111/ene.15613

**Published:** 2022-11-18

**Authors:** Chiara Pizzamiglio, Robert D. S. Pitceathly, Michael P. Lunn, Stefen Brady, Fabiola De Marchi, Lucia Galan, Jeannine M. Heckmann, Alejandro Horga, Maria J. Molnar, Acary S. B. Oliveira, Wladimir B. V. R. Pinto, Guido Primiano, Ernestina Santos, Benedikt Schoser, Serenella Servidei, Paulo V. Sgobbi Souza, Vishnu Venugopalan, Michael G. Hanna, Mazen M. Dimachkie, Pedro M. Machado, for the Neuromuscular Diseases and COVID‐19 Study Group, Albert Lim, Amar Elsaddig, Ana Juanatey, Ana Romeiro, Andreas Themistocleous, Annamaria Kiss‐Csenki, Antonio Guerrero Sola, Anuja Patil, Ashish Duggal, Carolyn Gabriel, Charles Marshall, Christopher Record, Claire Allen, David Bearden, DeviPriya Rathna Sabapathi, Dileep R, Domizia Vecchio, Edward Newman, Edwin Eshun, Eng C. Foo, Enrico Bugiardini, Georgina Burke, Gita Ramdharry, Gràinne S. Gorman, Guru Kumar, Harri Sivasathiaseelan, Igor Braga Farias, Izelle Smuts, James Holt, Jan T. Groothuis, Jane Pritchard, Jasmine Wall, Josep Gamez, K. J. S. Shakthi, Kate Wannop, Kathryn Brennan, Lillian Saavedra, Lisa Clayton, Liz Househam, Mariola Skorupinska, Matilde Laura, Matteo Ciocca, Maya Zosmer, Megha Dhamne, Michelangelo Mancuso, Mirian Janssen, Olimpia Musumeci, Olivia Price, Patrick F. Chinnery, Philip Ambrose, Puja R. Mehta, Rhys H. Thomas, Rita Horvath, Robert McFarland, Ross Nortley, Ross W. Paterson, Ruth Geraldes, Ryan Keh, Saara Neshuku, Sandhya Sasidharan, Sarath Menon R, Sharika Raga, Simon Rinaldi, Sireesha Yareeda, Soaham Desai, Sridharan Ramaratnam, Stephen Keddie, Taylor Watson‐Fargie, Teresinha Evangelista, Valeria Sansone, Victoria Nesbitt, William L. Macken, Yavuz Oktay

**Affiliations:** ^1^ Department of Neuromuscular Diseases UCL Queen Square Institute of Neurology London UK; ^2^ NHS Highly Specialised Service for Rare Mitochondrial Disorders, Queen Square Centre for Neuromuscular Diseases The National Hospital for Neurology and Neurosurgery London UK; ^3^ Department of Neurology John Radcliffe Hospital Oxford UK; ^4^ Department of Neurology and ALS Centre, Translational Medicine University of Piemonte Orientale, Maggiore Della Carità Hospital Novara Italy; ^5^ Neuromuscular Diseases Unit, Department of Neurology Hospital Clínico San Carlos and Instituto de Investigación Sanitaria San Carlos (IdISSC) Madrid Spain; ^6^ Division of Neurology, Department of Medicine University of Cape Town Cape Town South Africa; ^7^ Institute of Genomic Medicine and Rare Disorders Semmelweis University Budapest Hungary; ^8^ Division of Neuromuscular Diseases, Department of Neurology and Neurosurgery Federal University of São Paulo (UNIFESP) São Paulo Brazil; ^9^ Neurophysiopathology Unit Fondazione Policlinico Universitario A. Gemelli, IRCCS Rome Italy; ^10^ Department of Neuroscience Università Cattolica del Sacro Cuore Rome Italy; ^11^ Department of Neurology Centro Hospitalar Universitario do Porto, Hospital de Santo Antonio Oporto Portugal; ^12^ Department of Neurology, LMU Klinikum Friedrich‐Baur‐Institute, Ludwig‐Maximilians‐University Munich Munich Germany; ^13^ Department of Neurology All India Institute of Medical Sciences New Delhi India; ^14^ Department of Neurology University of Kansas Medical Centre Kansas City Kansas USA

**Keywords:** COVID‐19, Guillain–Barré syndrome, mitochondrial disease, neuromuscular diseases, outcome

## Abstract

**Background and purpose:**

Clinical outcome information on patients with neuromuscular diseases (NMDs) who have been infected with SARS‐CoV‐2 is limited. The aim of this study was to determine factors associated with the severity of COVID‐19 outcomes in people with NMDs.

**Methods:**

Cases of NMD, of any age, and confirmed/presumptive COVID‐19, submitted to the International Neuromuscular COVID‐19 Registry up to 31 December 2021, were included. A mutually exclusive ordinal COVID‐19 severity scale was defined as follows: (1) no hospitalization; (2) hospitalization without oxygenation; (3) hospitalization with ventilation/oxygenation; and (4) death. Multivariable ordinal logistic regression analyses were used to estimate odds ratios (ORs) for severe outcome, adjusting for age, sex, race/ethnicity, NMD, comorbidities, baseline functional status (modified Rankin scale [mRS]), use of immunosuppressive/immunomodulatory medication, and pandemic calendar period.

**Results:**

Of 315 patients from 13 countries (mean age 50.3 [±17.7] years, 154 [48.9%] female), 175 (55.5%) were not hospitalized, 27 (8.6%) were hospitalized without supplemental oxygen, 91 (28.9%) were hospitalized with ventilation/supplemental oxygen, and 22 (7%) died. Higher odds of severe COVID‐19 outcomes were observed for: age ≥50 years (50–64 years: OR 2.4, 95% confidence interval [CI] 1.33–4.31; >64 years: OR 4.16, 95% CI 2.12–8.15; both vs. <50 years); non‐White race/ethnicity (OR 1.81, 95% CI 1.07–3.06; vs. White); mRS moderately severe/severe disability (OR 3.02, 95% CI 1.6–5.69; vs. no/slight/moderate disability); history of respiratory dysfunction (OR 3.16, 95% CI 1.79–5.58); obesity (OR 2.24, 95% CI 1.18–4.25); ≥3 comorbidities (OR 3.2, 95% CI 1.76–5.83; vs. ≤2; if comorbidity count used instead of specific comorbidities); glucocorticoid treatment (OR 2.33, 95% CI 1.14–4.78); and Guillain–Barré syndrome (OR 3.1, 95% CI 1.35–7.13; vs. mitochondrial disease).

**Conclusions:**

Among people with NMDs, there is a differential risk of COVID‐19 outcomes according to demographic and clinical characteristics. These findings could be used to develop tailored management strategies and evidence‐based recommendations for NMD patients.

## INTRODUCTION

The coronavirus 2019 (COVID‐19) pandemic caused by severe acute respiratory syndrome coronavirus 2 (SARS‐CoV‐2) is one of the deadliest pandemics in history [1]. Factors associated with a severe outcome in the general population are older age and presence of underlying medical comorbidities, including chronic neurological disorders [2, 3]. However, more than 2 years after the start of the pandemic, there is still a paucity of data regarding risk and outcome of COVID‐19 in people with neuromuscular diseases (NMDs), especially considering the wide range of different diagnoses and disability burden, even in people with the same neuromuscular condition. COVID‐19 has the potential to severely affect patients with NMDs due to the possible involvement of respiratory muscles, the presence of comorbid diseases, and the use of immunosuppressive or immunomodulatory treatments [4]. General guidelines have been developed to inform and guide neuromuscular specialists during the COVID‐19 pandemic [5, 6]. The risk of severe COVID‐19 has been defined as moderate to high for most NMDs and people with these conditions were advised to follow rigid precautions to avoid the infection [6]. However, lockdown measures to suppress the COVID‐19 pandemic and protect vulnerable individuals, need to be balanced with the potential negative impact of confinement measures on neuromuscular, cardiovascular, metabolic and psychological health [7, 8].

To date there have been few studies that have investigated COVID‐19 outcomes and factors associated with these outcomes in patients with NMDs, and most of them have looked at small numbers of subjects and/or a single NMD [9, 10, 11, 12, 13, 14, 15, 16]. NMDs are often rare conditions when considered individually, and currently there is a knowledge gap regarding the clinical outcome of COVID‐19 in this patient group. Filling this gap could help clinicians in stratifying risks and in tailoring management for this vulnerable patient population.

The aim of this study was to determine factors associated with the severity of COVID‐19 outcomes in patients with NMDs.

## MATERIALS AND METHODS

### Data source

Data from the International Neuromuscular COVID‐19 Database were used. This registry was established by the Queen Square Centre for Neuromuscular Diseases at University College London (UCL), London, United Kingdom, and collects COVID‐19 cases in patients with NMDs submitted by physicians worldwide using an online portal (https://www.ucl.ac.uk/centre‐for‐neuromuscular‐diseases/news/2020/may/international‐neuromuscular‐covid‐19‐database) [17, 18, 19]. The survey was originally designed to capture COVID‐19 symptoms and outcomes for all NMDs, and is clinician‐reported: healthcare professionals caring for paediatric or adult neuromuscular patients voluntarily report cases to the registry. Physician data include name, specialty, hospital, city and country, and email address. The recruiting clinician entering data into the database could either have been the patient's primary COVID‐19 carer or could have been consulted about the patient, or the patient could be under the physician's care who then became aware he/she had COVID‐19. The database collects anonymized patient data only. The Neuromuscular COVID‐19 Registry data are securely stored at UCL in the United Kingdom and UCL is the data controller and data processor under General Data Protection Regulation. The UK Health Research Authority was consulted and advised that this project was considered to be a research database and that it did not require review by a National Health Service (NHS) Research Ethics Committee. The project was submitted as a ‘Service Evaluation’ to the Clinical Audit and Quality Improvement Subcommittee (CAQISC). There was no requirement for patient consent. Local guidelines were followed for non‐UK sites and this position was consistent through the non‐UK sites.

### Primary outcome

The primary outcome of interest of the present study was COVID‐19 outcome, assessed by use of an ordinal COVID‐19 severity scale with four mutually exclusive categories: (1) no hospitalization; (2) hospitalization without supplemental oxygen; (3) hospitalization with any supplemental oxygen, mechanical ventilation or extracorporeal membrane oxygenation (ECMO); and (4) death.

### Study participants

Subjects with an established pre‐existing or new (including potentially triggered/unmasked by SARS‐CoV‐2) genetic or acquired NMD, of any age, could be included. Physicians were asked to complete the database regardless of the severity of COVID‐19 and the NMD. A minimum of seven days between the COVID‐19 diagnosis and the registration into the database was required, as providers were asked to observe the outcome of the infection before submitting the case. The physician had to indicate if the diagnosis of COVID‐19 was confirmed (detection of SARS‐CoV‐2 nucleic acid or antigen), probable (evidence of clinical features of COVID‐19 plus a close contact with a confirmed COVID‐19 case in the 14 prior days, or radiological evidence of COVID‐19‐compatible lesions) or possible (evidence of clinical features only), based on the European Centre for Disease Prevention and Control (ECDC) definition [20].

### Other data collected

Case information included country of recruitment, age, sex, race/ethnicity, smoking status, NMD diagnosis, baseline functional status [21], comorbidities, medications at the time of COVID‐19 diagnosis, and outcome of infection (as reported in the ‘primary outcome’ section of this paper). For the survivors, information about ‘fully recovered’ or ‘recovered with sequelae’ status was collected. Baseline functional status was assessed using the modified Rankin scale (mRS), a six‐point disability scale initially applied to stroke patients but also proven valid in patients with NMDs [22]. The comorbidity ‘respiratory dysfunction’ included pre‐existing chronic obstructive pulmonary disease (COPD), asthma, interstitial lung disease, obstructive sleep apnoea, restrictive lung disease, and tracheostomy. The NMD was classified as: anterior horn cell disorder, neuropathy, neuromuscular junction disorder, muscle disorder. Exposure to immunomodulatory or immunosuppressive treatment at the time of COVID‐19 diagnosis was categorized as: glucocorticoids (prednisone‐equivalent dose specified); non‐biological drugs (hydroxychloroquine, chloroquine, azathioprine, cyclophosphamide, cyclosporine, leflunomide, methotrexate, mycophenolate mofetil/mycophenolic acid, sulfasalazine, tacrolimus, Janus kinase [JAK] inhibitors); and biologic drugs (abatacept, belimumab, CD‐20 inhibitors, interleukin [IL]‐1 inhibitors, IL‐6 inhibitors, IL‐12/IL‐23 inhibitors, IL‐17 inhibitors, tumour necrosis factor inhibitors). Two calendar periods according to the date of COVID‐19 diagnosis were established: on/before 15 June 2020, and after 15 June 2020, having as an arbitrary cut‐off the date of announcement of the RECOVERY trial glucocorticoid data [23].

### Statistics

Continuous data are expressed as mean ± standard deviation (SD) and categorical data as number and percentage (%). Multivariable ordinal logistic regression using the proportional odds model was used for the analysis of data, and odds ratios (ORs) and 95% confidence intervals (CIs) were estimated. In ordinal regression analysis, the effect size of a categorical predictor gives the chance in odds of being one level higher on the ordinal COVID‐19 severity scale compared to the reference category of the predictor variable. Patients with incomplete outcome data were excluded. Missing data from other variables were assumed to be missing at random and multiple imputation was performed to obtain a pooled estimate for ethnicity. An overall model included age group, sex, race/ethnicity, the four most common comorbidities, NMD diagnosis, mRS score, glucocorticoid and immunomodulatory treatment at the time of COVID‐19 diagnosis, and the pandemic calendar period.

Only NMDs with more than 20 subjects reported were analyzed separately. The mRS was dichotomized with the adopted cut‐off being the ability to walk independently. The most common comorbidities were tested and considered as binary variables (respiratory dysfunction, hypertension or other cardiovascular disease, diabetes, obesity).

An alternative variable (‘number of comorbidities’) representing the comorbidity burden was tested in an alternative model, considering the following comorbidities: respiratory dysfunction; hypertension or other cardiovascular disease; diabetes; obesity; cerebrovascular disease; pulmonary hypertension; chronic renal insufficiency; cancer; primary immunodeficiency; human immunodeficiency virus (HIV) infection; inflammatory bowel disease; liver disease; other non‐neuromuscular neurological disease; psychiatric condition; rheumatological disease; psoriasis; dysphagia; and enteral nutrition.

Sensitivity analyses were performed to assess the robustness of our results. A model excluding patients with Guillain–Barré syndrome (GBS), as these represented all new NMD diagnoses with normal or almost normal baseline (pre‐COVID‐19) functional status, was tested.

A *p* value <0.05 was taken to indicate statistical significance. Analyses were performed using SPSS v. 25.0 for Windows (SPSS Inc.).

The data that support the findings of this study are available from the corresponding author, upon reasonable request and justification, and approval from a steering committee.

## RESULTS

### Patient characteristics

From 1 May 2020 to 31 December 2021, 323 subjects with NMD were included in the database. Eight subjects without complete outcome information were excluded and 315 subjects were analysed. The demographic and clinical characteristics of the 315 subjects are shown in Table 1. Subjects were recruited from 13 countries, with most cases from the United Kingdom (107, 34%). The mean age was 50.3 (±17.7) years. The majority of subjects were male (161, 51.1%), and White (201, 66.6%). The only missing data were for race/ethnicity (13, 4.1%) and smoking status (25, 7.9%).

**TABLE 1:** Demographic and clinical characteristics of neuromuscular disease patients diagnosed with COVID‐19 (*N* = 315)

Characteristic	
Country of recruitment, *n* (%)	
United Kingdom	107 (34)
Brazil	50 (15.9)
Spain	37 (11.7)
Italy	36 (11.4)
South Africa	26 (8.3)
United States	26 (8.3)
India	12 (3.8)
Germany	6 (1.9)
Hungary	6 (1.9)
Portugal	5 (1.6)
Netherlands	2 (0.6)
Finland	1 (0.3)
Namibia	1 (0.3)
Sex at birth, *n* (%)	
Female	154 (48.9)
Male	161 (51.1)
Age group, *n* (%)	
<50 years	133 (42.2)
50–64 years	113 (35.9)
>64 years	69 (21.9)
Mean (± SD) age, years	50.3 (±17.7)
Race/ethnicity (*N* = 302), *n* (%)	
White	201 (66.6)
Latin American	46 (15.2)
Black	27 (8.9)
South Asian	23 (7.6)
East Asian	4 (1.3)
Arab	1 (0.3)
Neuromuscular disease diagnosis, *n* (%)	
(A) Anterior horn cell disorders	24 (7.6)
*Genetic*	
Spinal muscular atrophy	3 (1)
Riboflavin transporter deficiency	1 (0.3)
*Acquired*	
Amyotrophic lateral sclerosis	17 (5.4)
Post‐polio syndrome	3 (1)
(B) Neuropathies	100 (31.8)
*Genetic*	
Hereditary motor and sensory neuropathy	10 (3.2)
Amyloid neuropathy	3 (1)
Acute hepatic porphyria	2 (0.6)
*Acquired*	
Guillain–Barré syndrome	50 (15.9)
Chronic inflammatory demyelinating polyneuropathy	8 (2.5)
Multifocal motor neuropathy	4 (1.3)
Mononeuritis multiplex	3 (1)
Paraproteinemic neuropathy	3 (1)
Small‐fibre neuropathy	2 (0.6)
Autonomic neuropathy	1 (0.3)
Granulomatous neuropathy	1 (0.3)
Miller Fisher syndrome	1 (0.3)
Neuralgic amyotrophy	1 (0.3)
*Other*: Idiopathic neuropathy	11 (3.5)
(C) Neuromuscular junction disorders	63 (20)
*Genetic*	
Congenital myasthenic syndrome	5 (1.6)
*Acquired*	
Myasthenia gravis	56 (17.8)
Lambert‐Eaton myasthenic syndrome	2 (0.6)
(D) Muscle disorders	128 (40.6)
*Genetic*	
Mitochondrial disease	81 (25.7)
Limb girdle muscular dystrophy	9 (2.9)
Myotonic dystrophy	6 (1.9)
Congenital myopathy	5 (1.6)
X‐linked muscular dystrophy (Duchenne/Becker)	3 (1)
Facioscapulohumeral muscular dystrophy	2 (0.6)
Glycogen storage disease	2 (0.6)
Myofibrillar myopathy	1 (0.3)
Skeletal muscle channelopathy	1 (0.3)
Tubular aggregate myopathy	1 (0.3)
*Acquired*	
Idiopathic inflammatory myopathy	14 (4.4)
Acute rhabdomyolysis	3 (0.3)
Neuromuscular disease, *n* (%)	
New diagnosis^a^	60 (19)
Pre‐existing diagnosis	255 (81)
Baseline functional status (mRS), *n* (%)	
0. No symptoms	91 (28.9)
1. No significant disability	102 (32.4)
2. Slight disability	38 (12.1)
3. Moderate disability	26 (8.3)
4. Moderately severe disability	49 (15.6)
5. Severe disability	9 (2.9)
Most common comorbidities, *n* (%)	
Hypertension or other cardiovascular disease	93 (29.5)
Respiratory dysfunction^b^	69 (21.9)
Diabetes	55 (17.5)
Obesity (BMI ≥30 kg/m^2^)	52 (16.5)
Smoking status (*N* = 290), *n* (%)	
Ever	59 (18.7)
Never	231 (79.7)
Immunosuppressive or immunomodulatory treatment at the time of COVID‐19 diagnosis	
Glucocorticoids, *n* (%)	63 (20)
Prednisone‐equivalent dose, mean (± SD) mg/day	16.9 (± 13.6)
Other immunosuppressive/immunomodulatory treatment^c^, *n* (%)	61 (19.4)
Outcome of COVID‐19 infection, *n* (%)	
Not hospitalized	175 (55.5)
Hospitalized with no oxygenation	27 (8.6)
Hospitalized with ventilation/oxygenation	91 (28.9)
Death	22 (7)
Mean days from COVID‐19 onset to death (± SD)	15.6 (± 18.8)
Pandemic calendar period, *n* (%)	
1 January 2020 until 15 June 2020 (early)	98 (31.1)
16 June 2020 until 31 December 2021 (late)	217 (68.9)

Abbreviations: BMI, body mass index; ECMO, extracorporeal membrane oxygenation; mRS, modified Rankin Scale; SD, standard deviation.

^a^ Guillain–Barré syndrome (*n* = 50), idiopathic neuropathy (*n* = 3), mononeuritis multiplex (*n* = 2), acute rhabdomyolysis (*n* = 1), idiopathic inflammatory myopathy (*n* = 1), Miller Fisher syndrome (*n* = 1), myasthenia gravis (*n* = 1), small‐fibre neuropathy (*n* = 1).

^b^ Respiratory dysfunction includes: chronic obstructive pulmonary disease, asthma, interstitial lung disease, obstructive sleep apnoea, restrictive lung disease, and tracheostomy.

^c^ Azathioprine (*n* = 17), intravenous immunoglobulin (*n* = 14), methotrexate (*n* = 11), cyclosporine (*n* = 8), mycophenolate mofetil (*n* = 5), tacrolimus (*n* = 3), cyclophosphamide (*n* = 1), leflunomide (*n* = 1), rituximab (*n* = 1), thalidomide (*n* = 1). The sum exceeds the total of 61 due to patients under multiple treatments.

Regarding the NMD diagnosis, myopathy was present in 128 (40.6%) subjects, followed by neuropathy (100, 31.8%), neuromuscular junction disorder (63, 20%), and anterior horn cell disorder (24, 7.6%). Only mitochondrial disease (81, 25.7%), myasthenia gravis (56, 17.8%) and GBS (50, 15.9%) had more than 20 cases reported, with the remaining diseases being collapsed in the category ‘other’ (128, 40.6%). In 60 cases (19%) the NMD was a new diagnosis, including all 50 cases of GBS.

The most common comorbidities were hypertension or other cardiovascular disease (93, 29.5%), respiratory dysfunction (69, 21.9%), diabetes (55, 17.5%), and obesity (52, 16.5%). Fifty‐nine subjects (18.7%) were current or former smokers. Baseline functional status according to the mRS was as follows: no symptoms in 91 subjects (28.9%), no significant disability in 102 (32.4%), slight disability in 38 (12.1%), moderate disability in 26 (8.3%), moderately severe disability in 49 (15.6%) and severe disability in nine (2.9%).

### Outcome of COVID‐19

Of 315 patients, 175 (55.5%) were not hospitalized, 27 (8.6%) were hospitalized without supplemental oxygen, 91 (28.9%) were hospitalized and required ventilation or supplemental oxygen, and 22 (7%) died. A total of 198 subjects (62.9%) fully recovered, while 95 (30.2%) recovered with sequelae. The number of days between onset of COVID‐19 symptoms and resolution, or death, were 28.5 (±27.9) and 15.6 (±18.8), respectively. Most cases had a confirmed diagnosis of COVID‐19 according to the ECDC definition (268, 85.1%), while 16 (5.1%) were probable and 31 (9.8%) possible cases. Symptoms of COVID‐19 were present in 293 cases (93%), with the most common being fever (196, 62.2%), persistent cough (178, 56.5%), fatigue (165, 52.4%), shortness of breath (133, 42.2%), myalgia (111, 35.2%), headache (108, 34.3%), anosmia/hyposmia (89, 28.3%), and dysgeusia (65, 20.6%). Considering the pandemic calendar period, 98 subjects (31.1%) had COVID‐19 on/before 15 June 2020, while 217 (68.9%) had COVID‐19 after 15 June 2020.

**FIGURE 1:**
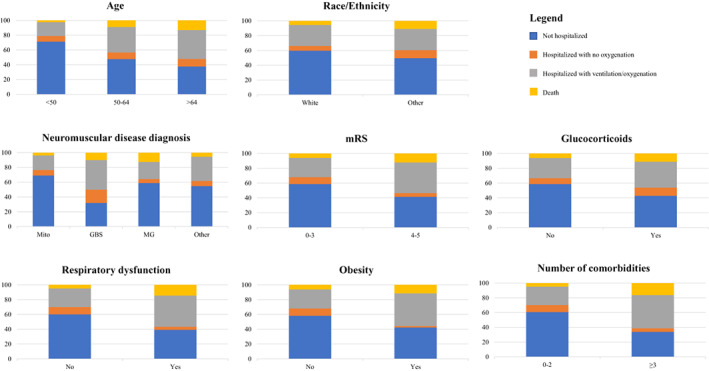
Distribution of COVID‐19 outcomes for the more significant risk factors. GBS, Guillain–Barré syndrome; Mito, mitochondrial disease; MG, myasthenia gravis; mRS, modified Rankin Scale.

The demographic and clinical characteristics of subjects with NMDs and COVID‐19, stratified by outcome severity, are available in Table 2 and Figure 1.

**TABLE 2:** Demographic and clinical characteristics of neuromuscular disease patients diagnosed with COVID‐19 stratified by ordinal severity outcome

	Not hospitalized (*n* = 175)	Hospitalized with no oxygenation (*n* = 27)	Hospitalized with ventilation/oxygenation (*n* = 91)	Death (*n* = 22)
Sex at birth, *n* (%)				
Female	93 (53.1)	14 (51.9)	40 (44)	7 (31.8)
Male	82 (46.9)	13 (48.1)	51 (56)	15 (68.2)
Age group, *n* (%)				
<50 years	95 (54.3)	10 (37)	25 (27.5)	3 (13.6)
50–64 years	54 (30.9)	10 (37)	39 (42.9)	10 (45.5)
>64 years	26 (14.9)	7 (25.9)	27 (29.7)	9 (40.9)
Race/ethnicity (*N* = 302), *n* (%)				
White	120 (70.6)	13 (54.2)	57 (66.3)	11 (50)
Other^a^	50 (29.4)	11 (45.8)	29 (33.7)	11 (50)
Neuromuscular disease diagnosis, *n* (%)				
Mitochondrial disease	56 (32)	6 (22.2)	16 (17.6)	3 (13.6)
GBS	16 (9.1)	9 (33.3)	20 (22)	5 (22.7)
Myasthenia gravis	33 (18.9)	3 (11.1)	13 (14.3)	7 (31.8)
Other^b^	70 (40)	9 (33.3)	42 (46.2)	7 (31.8)
Baseline functional status (mRS), *n* (%)				
0 to 3^c^	151 (86.3)	24 (88.9)	67 (73.6)	15 (68.2)
4 and 5^c^	24 (13.7)	3 (11.1)	24 (26.4)	7 (31.8)
Most common comorbidities, *n* (%)				
Respiratory dysfunction^d^	27 (15.4)	3 (11.1)	29 (31.9)	10 (45.5)
Hypertension or other CVD	42 (24)	6 (22.2)	34 (37.4)	11 (50)
Obesity (BMI ≥30 kg/m^2^)	22 (12.6)	1 (3.7)	23 (25.3)	6 (27.3)
Diabetes	28 (16)	3 (11.1)	20 (22)	4 (18.2)
Number of comorbidities, *n* (%)				
0 to 2 comorbidities	154 (88)	24 (88.9)	63 (69.2)	12 (54.5)
≥3 comorbidities	21 (12)	3 (11.1)	28 (30.8)	10 (45.5)
Smoking status (*N* = 290), *n* (%)				
Never smoker	133 (81.6)	23 (92)	59 (70.2)	16 (88.9)
Ever smoker	30 (18.4)	2 (8)	25 (29.8)	2 (11.1)
Immunomodulatory treatment at the time of COVID‐19 diagnosis, *n* (%)				
Immunomodulatory treatment^e^	32 (18.3)	5 (18.5)	17 (18.7)	7 (31.8)
Glucocorticoids	27 (15.4)	7 (25.9)	22 (24.2)	7 (31.8)
Pandemic calendar period, *n* (%)				
1 January 2020 until 15 June 2020 (early)	43 (24.6)	12 (44.4)	40 (44)	3 (13.6)
16 June 2020 until 31 December 2021 (late)	132 (75.4)	15 (55.6)	51 (56)	19 (86.4)

Abbreviations: BMI, body mass index; CVD, cardiovascular disease; GBS, Guillain–Barré syndrome; mRS, modified Rankin Scale.

^a^ Latin American (*n* = 46); Black (*n* = 27); South Asian (*n* = 23); East Asian (*n* = 4); Arab (*n* = 1).

^b^ ‘Other’ neuromuscular diagnosis includes: acute hepatic porphyria (*n* = 2), acute rhabdomyolysis (*n* = 3), amyloid neuropathy (*n* = 3), amyotrophic lateral sclerosis (*n* = 17), autonomic neuropathy (*n* = 1), chronic inflammatory demyelinating polyneuropathy (*n* = 8), congenital myasthenic syndrome (*n* = 5), congenital myopathy (*n* = 5), facioscapulohumeral muscular dystrophy (*n* = 2), granulomatous neuropathy (*n* = 1), glycogen storage disease (*n* = 2), idiopathic inflammatory myopathy (*n* = 14), idiopathic neuropathy (*n* = 11), hereditary motor and sensory neuropathy (*n* = 10), Lambert‐Eaton myasthenic syndrome (*n* = 2), limb girdle muscular dystrophy (*n* = 9), Miller Fisher syndrome (*n* = 1), mononeuritis multiplex (*n* = 3), multifocal motor neuropathy (*n* = 4), myofibrillar myopathy (*n* = 1), myotonic dystrophy (*n* = 6), neuralgic amyotrophy (*n* = 1), paraproteinemic neuropathy (*n* = 3), post‐polio syndrome (*n* = 3), riboflavin transporter deficiency (*n* = 1), skeletal muscle channelopathy (*n* = 1), small‐fibre neuropathy (*n* = 2), spinal muscular atrophy (*n* = 3), tubular aggregate myopathy (*n* = 1), X‐linked muscular dystrophy (Duchenne/Becker) (*n* = 3).

^c^ 0: No symptoms; 1: No significant disability; 2: Slight disability; 3: Moderate disability; 4: Moderately severe disability; 5: Severe disability.

^d^ Respiratory dysfunction includes: chronic obstructive pulmonary disease, asthma, interstitial lung disease, obstructive sleep apnoea, restrictive lung disease, and tracheostomy.

^e^ Azathioprine (*n* = 17), intravenous immunoglobulin (*n* = 14), methotrexate (*n* = 11), cyclosporine (*n* = 8), mycophenolate mofetil (*n* = 5), tacrolimus (*n* = 3), cyclophosphamide (*n* = 1), leflunomide (*n* = 1), rituximab (*n* = 1), thalidomide (*n* = 1). The sum exceeds the total of 61 due to patients under multiple treatments.

### Multivariable ordinal regression analyses

In the multivariable ordinal regression model analysis (Table 3), older age (50–64 years, OR 2.4, 95% CI 1.33–4.31; >64 years, OR 4.16, 95% CI 2.12–8.15; both vs. <50 years), other race/ethnicity (Latin American, South Asian, Black, East Asian, Arab; OR 1.81, 95% CI 1.07–3.06; vs. White race/ethnicity), diagnosis of GBS (OR 3.1, 95% CI 1.35–7.13; vs. mitochondrial disease), moderately severe/severe disability according to the mRS (OR 3.02, 95% CI 1.6–5.69; vs. no/slight/moderate disability), history of respiratory dysfunction (OR 3.16, 95% CI 1.79–5.58; vs. no respiratory dysfunction), obesity (body mass index ≥ 30 kg/m^2^; OR 2.24, 95% CI 1.18–4.25; vs. no obesity), and glucocorticoid treatment at the time of COVID‐19 diagnosis (OR 2.33, 95% CI 1.14–4.78; vs. no glucocorticoid treatment) were associated with higher odds of more severe COVID‐19 outcomes.

**TABLE 3:** Multivariable ordinal regression model analysis of factors associated with more severe COVID‐19 outcomes in people with neuromuscular diseases

	Odds ratio (95% CI)	*p* value
Age group		
<50 years	Reference	–
50–64 years	2.4 (1.33 to 4.31)	**0.004**
>64 years	4.16 (2.12 to 8.15)	**<0.001**
Male (vs. female)	1.36 (0.83 to 2.21)	0.22
Other race/ethnicity^a^ (vs. White)	1.81 (1.07 to 3.06)	**0.03**
Comorbidities (present vs. absent)		
Respiratory dysfunction^b^	3.16 (1.79 to 5.58)	**<0.001**
Diabetes	1.05 (0.54 to 2.06)	0.88
Hypertension or other CVD	1.37 (0.78 to 2.39)	0.28
Obesity (BMI ≥30 kg/m^2^)	2.24 (1.18 to 4.25)	**0.01**
Neuromuscular diseases diagnoses		
Mitochondrial disease	Reference	–
GBS	3.1 (1.35 to 7.13)	**0.008**
Myasthenia gravis	0.88 (0.31 to 2.47)	0.81
Other^c^	0.85 (0.42 to 1.72)	0.66
mRS: score 4–5 (vs. 0–3)^d^	3.02 (1.6 to 5.69)	**0.001**
Treatments (present vs. absent)		
Glucocorticoid treatment at the time of COVID‐19 diagnosis	2.33 (1.14 to 4.78)	**0.02**
Immunomodulatory treatment at the time of COVID‐19 diagnosis	1.13 (0.55 to 2.3)	0.75
Pandemic calendar period		
1 January 2020 until 15 June 2020 (early)	Reference	
16 June 2020 until 31 December 2021 (late)	0.68 (0.4 to 1.15)	0.1

*Note*: Significant *p* values highlighted in bold.

Abbreviations: BMI, body mass index; CI, confidence interval; CVD, cardiovascular disease; GBS, Guillain–Barré syndrome; mRS, modified Rankin Scale.

^a^ Latin American; Black; South Asian; East Asian; Arab.

^b^ Chronic obstructive pulmonary disease, asthma, interstitial lung disease, obstructive sleep apnoea, restrictive lung disease, and tracheostomy.

^c^ ‘Other’ neuromuscular diagnosis includes: acute hepatic porphyria (*n* = 2), acute rhabdomyolysis (*n* = 3), amyloid neuropathy (*n* = 3), amyotrophic lateral sclerosis (*n* = 17), autonomic neuropathy (*n* = 1), chronic inflammatory demyelinating polyneuropathy (*n* = 8), congenital myasthenic syndrome (*n* = 5), congenital myopathy (*n* = 5), facioscapulohumeral muscular dystrophy (*n* = 2), granulomatous neuropathy (*n* = 1), glycogen storage disease (*n* = 2), idiopathic inflammatory myopathy (*n* = 14), idiopathic neuropathy (*n* = 11), hereditary motor and sensory neuropathy (*n* = 10), Lambert‐Eaton myasthenic syndrome (*n* = 2), limb girdle muscular dystrophy (*n* = 9), Miller Fisher syndrome (*n* = 1), mononeuritis multiplex (*n* = 3), multifocal motor neuropathy (*n* = 4), myofibrillar myopathy (*n* = 1), myotonic dystrophy (*n* = 6), neuralgic amyotrophy (*n* = 1), paraproteinemic neuropathy (*n* = 3), post‐polio syndrome (*n* = 3), riboflavin transporter deficiency (*n* = 1), skeletal muscle channelopathy (*n* = 1), small‐fibre neuropathy (*n* = 2), spinal muscular atrophy (*n* = 3), tubular aggregate myopathy (*n* = 1), X‐linked muscular dystrophy (Duchenne/Becker) (*n* = 3).

^d^ 0: No symptoms; 1: No significant disability; 2: Slight disability; 3: Moderate disability; 4: Moderately severe disability; 5: Severe disability.

When replacing the main comorbidities (respiratory dysfunction, diabetes, hypertension or other cardiovascular disease, obesity) by the number of comorbidities (as an overall measure of comorbidity burden), the overall results were similar to the main model, and the presence of ≥3 comorbidities was associated with higher odds of more severe COVID‐19 outcomes (OR 3.2, 95% CI 1.76–5.83; Table 4).

**TABLE 4:** Multivariable ordinal regression model analysis of factors associated with more severe COVID‐19 outcomes in people with neuromuscular diseases, replacing the four commonest comorbidities by the number of comorbidities

	Odds ratio (95% CI)	*p* value
Age group		
<50 years	Reference	–
50–64 years	2.31 (1.3 to 4.12)	**0.005**
>64 years	4.02 (2.09 to 7.72)	**<0.001**
Male (vs. female)	1.32 (0.81 to 2.13)	0.26
Other race/ethnicity^a^ (vs. White)	1.72 (1.03 to 2.89)	**0.04**
≥3 comorbidities (vs. 0–2)	3.2 (1.76 to 5.83)	**<0.001**
Neuromuscular diseases diagnoses		
Mitochondrial disease	Reference	–
GBS	3.17 (1.41 to 7.13)	**0.005**
Myasthenia gravis	1.17 (0.43 to 3.18)	0.76
Other^b^	0.9 (0.45 to 1.79)	0.76
mRS: score 4–5 (vs. 0–3)^c^	2.95 (1.56 to 5.58)	**0.001**
Treatments (present vs. absent)		
Glucocorticoid treatment at the time of COVID‐19 diagnosis	2.13 (1.05 to 4.32)	**0.04**
Immunomodulatory treatment at the time of COVID‐19 diagnosis	1 (0.49 to 2.01)	0.99
Pandemic calendar period		
1 January 2020 until 15 June 2020 (early)	Reference	
16 June 2020 until 31 December 2021 (late)	0.75 (0.45 to 1.24)	0.26

*Note*: Significant *p* values highlighted in bold.

Abbreviations: CI, confidence interval; GBS, Guillain–Barré syndrome; mRS, modified Rankin Scale.

^a^ Latin American; Black; South Asian; East Asian; Arab.

^b^ ‘Other’ neuromuscular diagnosis includes: acute hepatic porphyria (*n* = 2), acute rhabdomyolysis (*n* = 3), amyloid neuropathy (*n* = 3), amyotrophic lateral sclerosis (*n* = 17), autonomic neuropathy (*n* = 1), chronic inflammatory demyelinating polyneuropathy (*n* = 8), congenital myasthenic syndrome (*n* = 5), congenital myopathy (*n* = 5), facioscapulohumeral muscular dystrophy (*n* = 2), granulomatous neuropathy (*n* = 1), glycogen storage disease (*n* = 2), idiopathic inflammatory myopathy (*n* = 14), idiopathic neuropathy (*n* = 11), hereditary motor and sensory neuropathy (*n* = 10), Lambert‐Eaton myasthenic syndrome (*n* = 2), limb girdle muscular dystrophy (*n* = 9), Miller Fisher syndrome (*n* = 1), mononeuritis multiplex (*n* = 3), multifocal motor neuropathy (*n* = 4), myofibrillar myopathy (*n* = 1), myotonic dystrophy (*n* = 6), neuralgic amyotrophy (*n* = 1), paraproteinemic neuropathy (*n* = 3), post‐polio syndrome (*n* = 3), riboflavin transporter deficiency (*n* = 1), skeletal muscle channelopathy (*n* = 1), small‐fibre neuropathy (*n* = 2), spinal muscular atrophy (*n* = 3), tubular aggregate myopathy (*n* = 1), X‐linked muscular dystrophy (Duchenne/Becker) (*n* = 3).

^c^ 0: No symptoms; 1: No significant disability; 2: Slight disability; 3: Moderate disability; 4: Moderately severe disability; 5: Severe disability.

Associations remained unchanged when removing GBS cases (all of them new‐onset COVID‐19‐associated cases) from the analysis (Tables 5 and 6), with older age, presence of respiratory dysfunction, obesity, ≥3 comorbidities, moderately severe/severe disability according to the mRS, and glucocorticoid treatment at the time of COVID‐19 diagnosis all remaining associated with higher odds of more severe COVID‐19 outcomes.

**TABLE 5:** Multivariable ordinal regression model analysis of factors associated with more severe COVID‐19 outcomes in people with neuromuscular diseases, excluding the patients with Guillain–Barré syndrome (*n* = 265)

	Odds ratio (95% CI)	*p* value
Age group		
<50 years	Reference	–
50–64 years	2.69 (1.38 to 5.24)	**0.004**
>64 years	4.65 (2.22 to 9.73)	**<0.001**
Male (vs. female)	1.3 (0.78 to 2.29)	0.3
Other race/ethnicity^a^ (vs. White)	1.56 (0.86 to 2.84)	0.15
Comorbidities (present vs. absent)		
Respiratory dysfunction^b^	2.82 (1.54 to 5.19)	**0.001**
Diabetes	0.97 (0.48 to 1.99)	0.94
Hypertension or other CVD	1.57 (0.86 to 2.85)	0.14
Obesity (BMI ≥30 kg/m^2^)	2.37 (1.16 to 4.83)	**0.02**
Neuromuscular diseases diagnoses		
Mitochondrial disease	Reference	–
Myasthenia gravis	0.85 (0.3 to 2.42)	0.76
Other^c^	0.81 (0.39 to 1.66)	0.56
mRS: score 4–5 (vs. 0–3)^d^	2.74 (1.43 to 5.26)	**0.002**
Treatments (present vs. absent)		
Glucocorticoid treatment at the time of COVID‐19 diagnosis	2.41 (1.13 to 5.11)	**0.02**
Immunomodulatory treatment at the time of COVID‐19 diagnosis	1.12 (0.54 to 2.29)	0.77
Pandemic calendar period		
1 January 2020 until 15 June 2020 (early)	Reference	
16 June 2020 until 31 December 2021 (late)	0.5 (0.28 to 0.92)	**0.03**

*Note*: Significant *p* values highlighted in bold.

Abbreviations: BMI, body mass index; CI, confidence interval; CVD, cardiovascular disease; mRS, modified Rankin Scale.

^a^ Latin American; Black; South Asian; East Asian; Arab.

^b^ Chronic obstructive pulmonary disease, asthma, interstitial lung disease, obstructive sleep apnoea, restrictive lung disease, and tracheostomy.

^c^ ‘Other’ neuromuscular diagnosis includes: acute hepatic porphyria (*n* = 2), acute rhabdomyolysis (*n* = 3), amyloid neuropathy (*n* = 3), amyotrophic lateral sclerosis (*n* = 17), autonomic neuropathy (*n* = 1), chronic inflammatory demyelinating polyneuropathy (*n* = 8), congenital myasthenic syndrome (*n* = 5), congenital myopathy (*n* = 5), facioscapulohumeral muscular dystrophy (*n* = 2), granulomatous neuropathy (*n* = 1), glycogen storage disease (*n* = 2), idiopathic inflammatory myopathy (*n* = 14), idiopathic neuropathy (*n* = 11), hereditary motor and sensory neuropathy (*n* = 10), Lambert‐Eaton myasthenic syndrome (*n* = 2), limb girdle muscular dystrophy (*n* = 9), Miller Fisher syndrome (*n* = 1), mononeuritis multiplex (*n* = 3), multifocal motor neuropathy (*n* = 4), myofibrillar myopathy (*n* = 1), myotonic dystrophy (*n* = 6), neuralgic amyotrophy (*n* = 1), paraproteinemic neuropathy (*n* = 3), post‐polio syndrome (*n* = 3), riboflavin transporter deficiency (*n* = 1), skeletal muscle channelopathy (*n* = 1), small‐fibre neuropathy (*n* = 2), spinal muscular atrophy (*n* = 3), tubular aggregate myopathy (*n* = 1), X‐linked muscular dystrophy (Duchenne/Becker) (*n* = 3).

^d^ 0: No symptoms; 1: No significant disability; 2: Slight disability; 3: Moderate disability; 4: Moderately severe disability; 5: Severe disability.

**TABLE 6:** Multivariable ordinal regression model analysis of factors associated with more severe COVID‐19 outcomes in people with neuromuscular diseases (excluding patients with Guillain–Barré syndrome), and replacing the four commonest comorbidities by the number of comorbidities (*n* = 265)

	Odds ratio (95% CI)	*p* value
Age group		
<50 years	Reference	–
50–64 years	2.42 (1.26 to 4.67)	**0.008**
>64 years	4.5 (2.18 to 9.3)	**<0.001**
Male (vs. female)	1.29 (0.76 to 2.19)	0.35
Other race/ethnicity^a^ (vs. White)	1.6 (0.89 to 2.89)	0.12
≥3 comorbidities (vs. 0–2)	3.13 (1.67 to 5.87)	**<0.001**
Neuromuscular diseases diagnoses		
Mitochondrial disease	Reference	–
Myasthenia gravis	1.1 (0.4 to 3.07)	0.85
Other^b^	0.87 (0.43 to 1.76)	0.69
mRS: score 4–5 (vs. 0–3)^c^	2.67 (1.39 to 5.12)	**0.003**
Treatments (present vs. absent)		
Glucocorticoid treatment at the time of COVID‐19 diagnosis	2.28 (1.09 to 4.77)	**0.03**
Immunomodulatory treatment at the time of COVID‐19 diagnosis	0.99 (0.49 to 2)	0.97
Pandemic calendar period		
1 January 2020 until 15 June 2020 (early)	Reference	
16 June 2020 until 31 December 2021 (late)	0.62 (0.35 to 1.1)	0.1

*Note*: Significant *p* values highlighted in bold.

Abbreviations: CI, confidence interval; mRS, modified Rankin Scale.

^a^ Latin American; Black; South Asian; East Asian; Arab.

^b^ ‘Other’ neuromuscular diagnosis includes: acute hepatic porphyria (*n* = 2), acute rhabdomyolysis (*n* = 3), amyloid neuropathy (*n* = 3), amyotrophic lateral sclerosis (*n* = 17), autonomic neuropathy (*n* = 1), chronic inflammatory demyelinating polyneuropathy (*n* = 8), congenital myasthenic syndrome (*n* = 5), congenital myopathy (*n* = 5), facioscapulohumeral muscular dystrophy (*n* = 2), granulomatous neuropathy (*n* = 1), glycogen storage disease (*n* = 2), idiopathic inflammatory myopathy (*n* = 14), idiopathic neuropathy (*n* = 11), hereditary motor and sensory neuropathy (*n* = 10), Lambert‐Eaton myasthenic syndrome (*n* = 2), limb girdle muscular dystrophy (*n* = 9), Miller Fisher syndrome (*n* = 1), mononeuritis multiplex (*n* = 3), multifocal motor neuropathy (*n* = 4), myofibrillar myopathy (*n* = 1), myotonic dystrophy (*n* = 6), neuralgic amyotrophy (*n* = 1), paraproteinemic neuropathy (*n* = 3), post‐polio syndrome (*n* = 3), riboflavin transporter deficiency (*n* = 1), skeletal muscle channelopathy (*n* = 1), small‐fibre neuropathy (*n* = 2), spinal muscular atrophy (*n* = 3), tubular aggregate myopathy (*n* = 1), X‐linked muscular dystrophy (Duchenne/Becker) (*n* = 3).

^c^ 0: No symptoms; 1: No significant disability; 2: Slight disability; 3: Moderate disability; 4: Moderately severe disability; 5: Severe disability.

## DISCUSSION

This is the largest study collecting cases of COVID‐19 in patients with NMDs, with 315 cases collected across 13 countries. Previous large studies have shown that chronic neurological disorders were associated with increased mortality for COVID‐19 in the general population [2, 3], although data about subjects with NMDs were not discussed.

We have identified factors associated with higher odds of more severe COVID‐19 outcomes, including older age, non‐White race/ethnicity, higher disease burden with moderately severe/severe disability on the mRS, respiratory dysfunction, obesity, presence of more than three comorbidities, and glucocorticoid treatment at the time of COVID‐19 diagnosis.

Within the different NMDs, GBS was associated with higher odds of worse COVID‐19 severity, compared to mitochondrial disease (the most prevalent diagnosis in the registry). However, this association should be interpreted with caution because GBS is an acute disease often diagnosed in the hospital setting. Importantly, associations remained unchanged when patients with GBS were removed from the analyses.

Eight patients with pre‐existing chronic inflammatory demyelinating polyneuropathy (CIDP) were reported to the registry during the collection period. Interestingly, clinical exacerbation of CIDP in association with SARS‐CoV‐2 infection has been described in the literature as a consequence of cytokine hyperactivation triggered by the virus [24].

Myasthenia gravis has been associated with higher mortality from COVID‐19 compared to the general population [12, 25, 26]; this can be explained by the presence of lung dysfunction and concomitant glucocorticoid/immunosuppressive treatment. In our study, within the different NMDs, we have not found myasthenia gravis to be associated with higher odds of severe COVID‐19. This may be attributable to the lack of phenotypic and antibody characterization of this heterogeneous patient population.

In our study, non‐White race/ethnicity was associated with worse COVID‐19 outcomes. Similar findings have been already described in the general population and in a large group of patients with chronic rheumatic diseases [27, 28]. These data confirm the presence of racial disparities in COVID‐19 outcomes, with non‐White groups having increased risk of hospitalization/death. The reasons for this difference are still under debate and might include the presence of risk factors for exposure to SARS‐CoV‐2 and poor outcomes, such as comorbidities and social determinants of health [29].

Male patients have been found to be more severely affected by SARS‐CoV2 than female patients [30]. The reasons for that are multifactorial and might include differences in adaptive and innate immune response, sex hormones, and prevalence of certain comorbidities [31]. In this study, we could see a trend towards male subjects being more severely affected than female subjects (68.2% of those deceased vs. 31.8%; and 56% of those requiring ventilation vs. 44%), but results were not significant in the subsequent regression analysis. A possible explanation includes lack of study power, but also the increased prevalence of female subjects with certain neuromuscular conditions such as myasthenia gravis [32].

In our cohort, 44.1% (*n* = 139, only one deceased patient was not hospitalized) of subjects were hospitalized, and 3/4 of these needed oxygen therapy, mechanical ventilation or ECMO. The frequency of hospitalization of NMD patients was higher than previous reports in the general population, although this may reflect selection bias and the voluntary nature of data collection in the registry and should not be considered as being a representation of the true rate of hospitalization in patients with NMDs. Similar considerations apply to the observed death rate of 7%.

Most patients recovered from COVID‐19, however, long‐term sequelae were reported in 30.2% of subjects. Sequelae were predominantly observed in patients discharged from hospital (62%), in line with the study by Huang et al., showing that patients with severe illness had an increased risk of lung abnormalities, muscle weakness or fatigue, and psychiatric symptoms, even at 6 months from COVID‐19 symptom onset [33].

We found an association between the use of glucocorticoids before COVID‐19 diagnosis and worse COVID‐19 outcome. The same finding has been reported in large series of patients with myasthenia gravis [12, 26], rheumatic disease [34] and inflammatory bowel disease [35]. This does not contradict evidence showing the benefit of glucocorticoids in reducing mortality for severe COVID‐19, as the beneficial effect of dexamethasone/hydrocortisone is restricted to critically ill patients who were receiving oxygen or invasive mechanical ventilation at randomization [36, 37, 38]. Our results suggest the possibility of a worse outcome in autoimmune compared to hereditary disorders. Indeed, we report that subjects with GBS and subjects on glucocorticoids have higher odds of severe COVID‐19 outcome.

No association was found between pre‐existing immunomodulatory therapy and COVID‐19 outcome. Data from previous literature on patients with multiple sclerosis [39], myasthenia gravis [26] and rheumatic disease [34] show similar results, except for B‐cell‐depleting therapies. Therefore, guidelines generally suggest continuation of immunosuppressive medications at COVID‐19 diagnosis [14, 34, 40].

The degree of disability at baseline was associated with severe COVID‐19, with higher odds of worse outcome for those with moderately severe/severe disability (patients not able to walk independently) according to the mRS [22]. This further supports the concept that, in chronic diseases, disease burden is associated with COVID‐19 severity, as previously shown in patients with multiple sclerosis, and should be considered when counselling patients with NMD [39].

The presence and number of comorbidities is a well‐known risk factor for severe COVID‐19 [2, 3, 41] and, indeed, this was confirmed in patients with NMDs. In the general population, the strongest link with severe COVID‐19 was found for diabetes, hypertension, cardiovascular disease, COPD and obesity [42, 43]. In our cohort, the presence of respiratory dysfunction, including both non‐NMD and NMD‐associated pulmonary diseases, and obesity were associated with higher odds of worse COVID‐19 outcomes. Importantly, the overall comorbidity burden plays a role in COVID‐19 severity in our population, as demonstrated by the increased odds of worse outcomes in subjects with three or more comorbidities. In this cohort, we have also identified NMD‐specific factors associated with increased odds of severe COVID‐19, such as a high degree of disability at baseline (mRS score greater or equal to 4), GBS, and pre‐existing steroid treatment.

Strengths of this study include the fact it was the first global, large analysis of patients with NMDs and COVID‐19. These findings will enable risk stratification and appropriate management of patients with NMDs. However, our study has limitations. It was a cross‐sectional study with data added voluntarily to the registry; therefore, as selection bias may exist, the study cannot be used to comment on hospitalization, death rate, or the global prevalence of NMDs as it might not reflect the prevalence of the different NMDs in the general population. Information on SARS‐CoV‐2 variants across the study is also not available; however, data were collected from 1 May 2020 to 31 December 2021, when the original Wuhan strain, alpha, delta, and omicron variants were predominant. The database did not collect information about vaccination status; however, the change in public health measures and availability of effective treatments has been considered in the ‘pandemic calendar period’ variable. Further sensitivity analyses, taking into account different types of respiratory dysfunction, were also not possible, due to overlap of respiratory dysfunction categories and lack of granularity about the underlying respiratory disease. Similarly, due to the nature of this study, granular data about each NMD subgroup were not available, and numbers are small for some of the conditions, which may have affected the robustness of our estimates, and highlights the relevance of ongoing data collection, particularly for rarer disease subsets. Moreover, we caution against interpreting our estimates causally. There is likely unmeasured confounding dependent on the particularities of health systems and case reporting differences. We tried to address this by limiting the research questions to those that could be answered with this dataset and by accounting for potential confounders in our analyses.

In conclusion, older age, non‐White race/ethnicity, respiratory dysfunction, obesity, higher comorbidity burden, higher baseline disability, and glucocorticoid treatment, were associated with severe COVID‐19. Among the different NMDs, GBS was associated with higher odds of worse COVID‐19 outcomes. Our findings will help clinicians worldwide in developing tailored management strategies for patients with NMD during COVID‐19 waves or similar respiratory pandemics in future.

## AUTHOR CONTRIBUTIONS

Conception and design: Chiara Pizzamiglio, Robert D. S. Pitceathly, Pedro M. Machado. Acquisition and analysis of data: Chiara Pizzamiglio, Robert D. S. Pitceathly, Michael P. Lunn, Stefen Brady, Fabiola De Marchi, Lucia Galan, Jeannine M. Heckmann, Alejandro Horga, Maria J. Molnar^7^, Acary S. B. Oliveira, Wladimir B. V. R. Pinto, Guido Primiano, Ernestina Santos, Benedikt Schoser, Serenella Servidei, Paulo V. Sgobbi Souza, Vishnu Venugopalan, Michael G. Hanna, Mazen M. Dimachkie, Pedro M. Machado. Drafting of the manuscript or figures: Chiara Pizzamiglio, Pedro M. Machado. Neuromuscular Diseases and COVID‐19 Study Group members and affiliations are available in the Supplementary Information.

## CONFLICT OF INTEREST

Benedikt Schoser has received consulting/speaker's fees from Argenx, Amicus, Audentes, Avrobio, Dynacure, Sanofi‐Genzyme, Spark and Taysha. Mazen M. Dimachkie serves or has recently served as a consultant for Amazentis, ArgenX, Catalyst, Cello, Covance/Labcorp, CSL‐Behring, EcoR1, Janssen, Kezar, Medlink, Momenta, NuFactor, Octapharma, RaPharma/UCB, Roivant Sciences Inc, RMS Medical, Sanofi Genzyme, Shire Takeda, Scholar Rock, Spark Therapeutics, Third Rock, UCB Biopharma and UpToDate. Lucia Galan has received honoraria as a speaker and advisor from Akcea, Alnylam, Grunenthal, and Pfizer. Mazen M. Dimachkie has received research grants or contracts or educational grants from Alexion, Alnylam Pharmaceuticals, Amicus, Biomarin, Bristol‐Myers Squibb, Catalyst, Corbus, CSL‐Behring, FDA/OOPD, GlaxoSmithKline, Genentech, Grifols, Kezar, Mitsubishi Tanabe Pharma, MDA, the NIH, Novartis, Octapharma, Orphazyme, Ra Pharma/UCB, Sanofi Genzyme, Sarepta Therapeutics, Shire Takeda, Spark Therapeutics, the Myositis Association, UCB Biopharma / RaPharma, Viromed/Healixmith and TMA. Pedro M. Machado has received consulting/speaker's fees from Abbvie, BMS, Celgene, Eli Lilly, Galapagos, Janssen, MSD, Novartis, Orphazyme, Pfizer, Roche and UCB.

## Supporting information


Supplementary material S1

## Data Availability

Data are available upon reasonable request by qualified researchers who engage in rigorous, independent scientific research, and will be provided following review and approval by a steering committee of a detailed research proposal and statistical analysis plan and following execution of applicable data sharing agreements.
