# Supplementary material for: Factors associated with the severity of COVID‐19 outcomes in people with neuromuscular diseases: Data from the International Neuromuscular COVID‐19 Registry
Source: Eur J Neurol. 2022 Nov 18;30(2):399–412. doi: 10.1111/ene.15613 (PMC9874570; doi:10.1111/ene.15613)
Supplement: Supplementary file 1 — Supplementary material S1 [file ENE-30-399-s001.docx]

**Supplementary table**

**Group Name:** Neuromuscular Diseases and COVID-19 Collaborators

| **First Name and Middle Initial(s)** | **Last Name** | **Institution** | **Location (city, state/province, country)** |
| --- | --- | --- | --- |
| Albert | Lim | Royal Victoria Infirmary, The Newcastle upon Tyne Hospitals NHS Trust | Newcastle upon Tyne, UK |
| Amar | Elsaddig | Salford Royal Hospital, Northern care Alliance NHS Trust | Salford Royal, UK |
| Ana | Juanatey | Hospital de Zamora | Zamora, Spain |
| Ana | Romeiro | Wexham Park Hospital, Frimley Health NHS Trust | Slough, UK |
| Andreas | Themistocleous | John Radcliffe Hospital, Oxford University Hospitals NHS Trust | Oxford, UK |
| Annamaria | Kiss-Csenki | Basingstoke and North Hampshire Hospital, Hampshire Hospitals NHS Trust | Basingstoke, UK |
| Antonio | Guerrero Sola | Hospital Clinico San Carlos | Madrid, Spain |
| Anuja | Patil | KIMS Secunderabad | Hyderabad, India |
| Ashish | Duggal | G. B. Pant Institute of Postgraduate Medical Education and Research | New Delhi, India |
| Carolyn | Gabriel | St Mary's Hospital, Imperial College Healthcare NHS Trust | London, UK |
| Charles | Marshall | Royal London Hospital, Barts Health NHS Trust | London, UK |
| Christopher | Record | St George’s Hospital, St George’s University Hospitals NHS Trust | London, UK |
| Claire | Allen | Poole Hospital, University Hospitals Dorset NHS Trust | Poole, UK |
| David | Bearden | University of Rochester Medical Center | Rochester, USA |
| DeviPriya | Rathna Sabapathi | Southampton General Hospital, University Hospital Southampton NHS Trust | Southampton, UK |
| Dileep | R | Government Medical College | Thiruvananthapuram, India |
| Domizia | Vecchio | AOU Maggiore della Carità | Novara, Italy |
| Edward | Newman | Glasgow Royal Infirmary, NHS Greater Glasgow and Clyde | Glasgow, UK |
| Edwin | Eshun | National Hospital for Neurology and Neurosurgery, University College London Hospitals NHS Trust | London, UK |
| Eng C | Foo | Calderdale Royal Hospital, Calderdale and Huddersfield NHS Trust | Halifax, UK |
| Enrico | Bugiardini | National Hospital for Neurology and Neurosurgery, University College London Hospitals NHS Trust | London, UK |
| Georgina | Burke | Queen Alexandra Hospital, Portsmouth Hospitals University NHS Trust | Portsmouth, UK |
| Gita | Ramdharry | National Hospital for Neurology and Neurosurgery, University College London Hospitals NHS Trust | London, UK |
| Gràinne S | Gorman | Royal Victoria Infirmary, The Newcastle upon Tyne Hospitals NHS Trust | Newcastle upon Tyne, UK |
| Guru | Kumar | Darent Valley Hospital, Dartford and Gravesham NHS Trust | Dartford, UK |
| Harri | Sivasathiaseelan | Homerton Hospital, Homerton University Hospital NHS Trust | London, UK |
| Igor | Braga Farias | Division of Neuromuscular Diseases, Federal University of São Paulo (UNIFESP) | São Paulo, Brazil |
| Izelle | Smuts | University of Pretoria | Pretoria, South Africa |
| James | Holt | Countess of Chester Hospital, Cheshire and Wirral Partnership NHS Trust | Chester, UK |
| Jan T | Groothuis | Department of Rehabilitation, Radboud University Medical Center | Nijmegen, Netherlands |
| Jane | Pritchard | Charing Cross Hospital, Imperial College Healthcare NHS Trust | London, UK |
| Jasmine | Wall | Royal Preston Hospital, Lancashire Teaching Hospital NHS Trust | Preston, UK |
| Josep | Gamez | GMA Clinic. ERN EURO-NMD. Autonomous University of Barcelona | Barcelona, Spain |
| K J S | Shakthi | BGS Gleneagles Global Hospital | Bangalore, India |
| Kate | Wannop | King's College Hospital, King's College Hospital NHS Trust | London, UK |
| Kathryn | Brennan | University Hospital Wishaw, NHS Lanarkshire | Wishaw, UK |
| Lillian | Saavedra | Department of Neurology, University of Kansas Medical Centre | Kansas City, KS, USA |
| Lisa | Clayton | Royal London Hospital, Barts Health NHS Trust | London, UK |
| Liz | Househam | Derriford Hospital, University Hospitals Plymouth NHS Trust | Plymouth, UK |
| Mariola | Skorupinska | National Hospital for Neurology and Neurosurgery, University College London Hospitals NHS Trust | London, UK |
| Matilde | Laura | National Hospital for Neurology and Neurosurgery, University College London Hospitals NHS Trust | London, UK |
| Matteo | Ciocca | Charing Cross Hospital, Imperial College Healthcare NHS Trust | London, UK |
| Maya | Zosmer | North Middlesex University Hospital, North Middlesex University Hospital NHS Trust | London, UK |
| Megha | Dhamne | Dr. L H Hiranandani Hospital, Powai | Mumbai, India |
| Michelangelo | Mancuso | Department of Clinical and Experimental Medicine, Neurological Institute, University of Pisa | Pisa, Italy |
| Mirian | Janssen | Radboud University Medical Center | Nijmegen, Netherlands |
| Olimpia | Musumeci | AOU Policlinico G. Martino | Messina, Italy |
| Olivia | Price | Basildon Hospital, Mid and South Essex NHS Trust | Basildon, UK |
| Patrick F | Chinnery | Addenbrooke’s Hospital, Cambridge University Hospitals NHS Trust | Cambridge, UK |
| Philip | Ambrose | Queens Medical Centre, Nottingham University Hospitals NHS Trust | Nottingham, UK |
| Puja R | Mehta | King's College Hospital, King's College Hospital NHS Trust | London, UK |
| Rhys H | Thomas | Royal Victoria Infirmary, The Newcastle upon Tyne Hospitals NHS Trust | Newcastle upon Tyne, UK |
| Rita | Horvath | Addenbrooke’s Hospital, Cambridge University Hospitals NHS Trust | Cambridge, UK |
| Robert | McFarland | Royal Victoria Infirmary, The Newcastle upon Tyne Hospitals NHS Trust | Newcastle upon Tyne, UK |
| Ross | Nortley | Wexham Park Hospital, Frimley Health NHS Trust | Slough, UK |
| Ross W | Paterson | Darent Valley Hospital, Dartford and Gravesham NHS Trust | Dartford, UK |
| Ruth | Geraldes | Wexham Park Hospital, Frimley Health NHS Trust | Slough, UK |
| Ryan | Keh | Royal Preston Hospital, Lancashire Teaching Hospital NHS Trust | Preston, UK |
| Saara | Neshuku | Katutura State Hospital | Windhoek, Namibia |
| Sandhya | Sasidharan | Department of Neurology, University of Kansas Medical Centre | Kansas City, KS, USA |
| Sarath | Menon R | Sree Narayana Institute of Medical Sciences | Kochi, India |
| Sharika | Raga | Red Cross War Memorial Children's Hospital | Cape Town, South Africa |
| Simon | Rinaldi | John Radcliffe Hospital, Oxford University Hospitals NHS Trust | Oxford, UK |
| Sireesha | Yareeda | Nizam's Institute of Medical Sciences | Hyderabad, India |
| Soaham | Desai | Shree Krishna Hospital and Pramukhswami Medical College | Anand, India |
| Sridharan | Ramaratnam | The Nerve Centre | Chennai, India |
| Stephen | Keddie | National Hospital for Neurology and Neurosurgery, University College London Hospitals NHS Trust & Department of Neuromuscular Disease, Royal London Hospital, Barts and the Royal London NHS Trust | London, UK |
| Taylor | Watson-Fargie | Queen Elizabeth University Hospital, NHS Greater Glasgow and Clyde | Glasgow, UK |
| Teresinha | Evangelista | Sorbonne University, Pitié Salpêtrière Hospital, Assistance Public Hopitaux de Paris | Paris, France |
| Valeria | Sansone | Nemo Clinical Center | Milan, Italy |
| Victoria | Nesbitt | John Radcliffe Hospital, Oxford University Hospitals NHS Trust | Oxford, UK |
| William L | Macken | National Hospital for Neurology and Neurosurgery, University College London Hospitals NHS Trust | London, UK |
| Yavuz | Oktay | Izmir Biomedicine and Genome Center and Institute (IBG) | Izmir, Turkey |
